# Shrinking a large dataset to identify variables associated with increased risk of *Plasmodium falciparum* infection in Western Kenya

**DOI:** 10.1017/S0950268815000710

**Published:** 2015-04-16

**Authors:** M. TREMBLAY, J. S. DAHM, C. N. WAMAE, W. A. DE GLANVILLE, E. M. FÈVRE, D. DÖPFER

**Affiliations:** 1 Departments of Medicine and Pathobiological Sciences, School of Veterinary Medicine, University of Wisconsin-Madison, Madison, WI, USA; 2 Center for Microbiology Research, Kenya Medical Research Institute (KEMRI), Nairobi, Kenya; 3 School of Health Sciences, Mount Kenya University, Thika, Kenya; 4 Centre for Immunity, Infection and Evolution, Institute for Immunology and Infection Research, School of Biological Sciences, University of Edinburgh, Ashworth Laboratories, Edinburgh, UK; 5 International Livestock Research Institute, Nairobi, Kenya; 6 Institute of Infection and Global Health, University of Liverpool, Leahurst Campus, Neston, UK

**Keywords:** Cattle, data mining, Kenya, malaria, zoonotic diseases

## Abstract

Large datasets are often not amenable to analysis using traditional single-step approaches. Here, our general objective was to apply imputation techniques, principal component analysis (PCA), elastic net and generalized linear models to a large dataset in a systematic approach to extract the most meaningful predictors for a health outcome. We extracted predictors for *Plasmodium falciparum* infection, from a large covariate dataset while facing limited numbers of observations, using data from the People, Animals, and their Zoonoses (PAZ) project to demonstrate these techniques: data collected from 415 homesteads in western Kenya, contained over 1500 variables that describe the health, environment, and social factors of the humans, livestock, and the homesteads in which they reside. The wide, sparse dataset was simplified to 42 predictors of *P. falciparum* malaria infection and wealth rankings were produced for all homesteads. The 42 predictors make biological sense and are supported by previous studies. This systematic data-mining approach we used would make many large datasets more manageable and informative for decision-making processes and health policy prioritization.

## INTRODUCTION

With the increasing production and availability of large amounts of data, it is common to have datasets that cannot be analysed using traditional single-step approaches. For example, it is not advisable to build simple regression models from datasets that have thousands of variables or those that have incomplete data. Many different data-mining and statistical techniques are commonly employed individually to address these issues, but a systematic approach has not been developed to take advantage of multiple methods’ strengths and capacities. Our general objective is to apply imputation techniques, principal component analysis (PCA), elastic net and generalized linear models (GLM) in a systematic approach to extract the most meaningful predictors for a health outcome from a large covariate dataset while facing limited numbers of observations. The People, Animals, and their Zoonoses (PAZ) dataset will be used to demonstrate these techniques [1]. The PAZ project's goal is to explore the epidemiology and burden of a number of neglected zoonotic diseases in a sympatric population of animals and people. Currently, PAZ's only study site is in Western Kenya. The dataset contained variables that describe the health, environment, and social factors of the humans, livestock, and homesteads in which they reside. The specific aim of applying this protocol to the PAZ dataset is to develop and apply socioeconomic wealth indices and determine the best predictors of falciparum malaria infection exposure prevalence in individuals included in the PAZ dataset [2]. We hypothesize that these techniques can be used to develop a simplified dataset with the most meaningful predictors from a wide, sparse dataset. If successful, this systematic data-mining approach could make many large datasets more manageable and informative.

## MATERIALS AND METHODS

### Making a complete dataset

The dataset used in this study which originates from the PAZ project consist of questionnaire data from 416 rural homesteads and biological sampling data of 2113 humans and 983 cattle from these homesteads in the western Province of Kenya [1]. Homesteads determined to be outliers due to an extreme cattle–human ratio were excluded from the analysis.

All data analyses were performed using R version 3·0·1 [3]. A case of malaria was defined as a subject being positive for *Plasmodium falciparum* on thick or thin blood smears [4]. The homestead malaria prevalence was defined by:
1
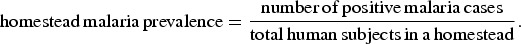


To prepare the dataset for statistical analysis, all categorical variables were expanded into binary dummy variables and edited until missing values were all coded as ‘NA’. The number of missing values was first calculated per dataset and frequency tables were used to examine the percent missingness per variable. Variables with >10% of values missing were removed from the dataset. This was important, because the deleted variables could not be determined to be ‘missing at random (MAR)’ due to the non-random approach to the data collection, and therefore keeping those variable in the dataset would have conflicted with the MAR prerequisite of multiple imputations [5].

After this new dataset was generated and further missingness was assumed to be at random, the remaining variables were subjected to piecewise multiple imputations by chained equations using the R package ‘mice’ [6, 7]. This package was selected due to its ability to handle both factor and continuous variables. After completing the imputation by ‘mice’, variables with missing values that could not be imputed were omitted from further statistical testing.

Frequency tables were created for all variables and data were analysed for uniformity. Variables where the most frequent value accounted for ⩾99% of the observations were removed to avoid variables without contrasts in the dataset. A range of such cut-off percentages for uniformity was evaluated and the 99% cut-off resulted in the most consistent removal of variables without contrasts across the dataset.

Variables denoting the number of individuals per homestead for cattle and humans were created to serve as denominators for calculating prevalences. For each numeric variable in the human and cattle dataset, the mean value across each homestead was calculated to subsequently allow the dataset to be merged by homestead number.

### Ethical considerations

Human data and samples collected in this study were collected following approval by the KEMRI Ethical Review Committee, SC#1701. Animal samples were collected following approval from the Roslin Institute Animal Welfare and Ethical Review Committee, AWA004. The Institutional Review Board (IRB) approved this study (IRB no. 2013-0072).

### Creating wealth indices using PCA

Because wealth is often a predictor of disease prevalence, selected asset and livestock variables descriptive of wealth or socioeconomic status were shrunk into one wealth ranking value per homestead [2]. Historically, asset-based wealth indices have been based on household assets, but because wealth in rural areas is often dependent upon livestock ownership and the ability to call on human assistance, compared to urban areas, in which wealth is often expressed in material possessions, two separate wealth rankings were created: one based on material assets (asset-based wealth ranking) and one based on a homestead's livestock (livestock-based wealth ranking) [2].

Both wealth indices were created using PCA, an ordination method commonly applied during wealth-indexing studies [8]. PCA converts a number of non-correlated variables into a number of orthogonal principal components (PCs) [9]. The first PC is the ordination of the variables that explains the most amount of variance, and each subsequent PC thereafter explains a decreasing amount of the variance. The starting subset of variables for each wealth index was selected from a previous study by Okell *et al*. that utilized a preliminary version of the same dataset with fewer homesteads [8]. All variables were formatted as numeric, and their respective minima were added to each variable set to assure non-negative values. The variables were scaled using the ‘scale()’ command in order to assure non-negative values in the dataset used for PCA, i.e. the overall minimum value of any observation was added to all values in the dataset.

Because highly correlated variables can skew a PCA analysis, a Pearson correlation matrix was used on both the asset-based and livestock-based variables to determine whether any two variables were highly correlated, in which case the biologically less relevant variable was removed. A correlation ⩾90% was used as our limit [10]. The PCA was run on both the asset-based and livestock-based variables separately [11]. Based on the first six PCs of each of the two PCAs, it was determined which subset of variables contributed more than expected to the explanation of the overall variance in the respective datasets. The PCAs were repeated for the selected subset of covariates. The respective first PCs of the outcomes were taken as the livestock-based and asset-based wealth indices.

To explore the validity of the livestock wealth index, a third wealth index was created based on real-world valuation of livestock holdings. Current market value for each category of the livestock evaluated was based on interviews with market traders in the study region and subsequently multiplied by the number of livestock in the respective livestock categories of the dataset [8]. The summation of these values yielded the total livestock value (TLV) for each homestead, which was used as a real-world approximation estimate for livestock wealth [8]:
2
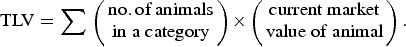


These wealth indices were merged with the final dataset by homestead. Since only 54% of the homesteads had cattle, the final dataset including the wealth indices was divided into two datasets for further analysis. Subset A was created from the homestead, human, and cattle variables containing only the 224 homesteads with cattle. Subset B was created using the homestead and human variables of all 415 homesteads only.

### Selecting predictors with elastic net and GLM

Regularized regression models are a commonly accepted method for selecting predictors from large data. The elastic net was created by combining the penalties of the lasso and ridge regularized regression methods. This combination allows for better performance when the number of variables (*p*) is greater than the observation count (*n*) and when groups of variables exist that are highly correlated while still resulting in a parsimonious model [12]. The number of variables selected is controlled by the alpha (*α)* parameter. The regression will more closely resemble a lasso regression or a ridge regression as α nears/approaches 1 or 0, respectively [12].

The glmnet package in R was used to fit the elastic-net regularization path for Poisson regression on homestead malaria prevalence for subsets A and B [13]. The model response was the count of malaria-positive cases in each homestead and an offset of the log of the total humans per homestead was used to model prevalence. A Poisson family was chosen since the response was a count. The cross-validation function (cv.glmnet) was used to find the best value of lambda (*λ*), the regularization parameter, and the number of folds was selected to be the number of observations (*n*) minus 1 (leave-one-out cross-validation). To select the best value of *α*, 50 iterations of 17 different *α* values between 0 and 1 were run and summarized. The *α* *value* that resulted in the lowest mean absolute error (MAE) was selected. The selected *λ* and *α* values were subsequently used for elastic-net variable selection using the glmnet function.

The variables selected by the elastic-net regularized penalized regression using non-zero coefficients were subsetted and included in a GLM using the glm package in R. Further variable selection was performed in a stepwise function based on Akaike's Information Criterion (AIC) using the step function. Both forward and backward directions were allowed [2]. To determine significance of covariates an error level, *α* = 0·05 was set. A model with only significant variables was desired so further backwards elimination was performed based on *P* value.

## RESULTS

### Making a complete dataset

Homestead 84 was considered an outlier due to a very high cattle–human ratio; therefore, all observations from homestead 84 (17 human subjects, 41 cattle) were excluded from the analysis. Eleven cattle and one human subject were removed because they did not have a homestead number recorded, 415 homesteads, 2095 humans and 931 cattle remained.

In the homestead dataset 2·81% (4753/168 905) of values were missing and there were 24/407 variables with >10% missingness. In the cattle dataset 16·95% (48 750/287 679) of values were missing and there were 78/309 variables with >10% missingness. In the human dataset 8·09% ( 111 810/1 382 700) of values were missing and there were 105/660 variables with >10% missingness. After the variables with >10% missing values were removed, 1169 variables remained. The number of variables left and removed per dataset is described in Table 1.

**Table 1.** Number of variables per dataset at each step

	Homestead	Human	Livestock
1. Starting number of variables	407	660	309
2. Number of variables removed due to >10% missingness	−24	−105	−78
3. Number of variables removed due to incomplete imputation	−18	−16	−2
4. Number of variables removed due to >99% uniformity	−93	−188	−97
5. Final number of variables	272	351	132

There were 677 values still missing in the cattle dataset (0·32%, 677/215 061), 14 742 values still missing in the human dataset (1·27%, 14 742/1 164 820) and 1296 values still missing in the homestead dataset (0·82%, 1295/158 945) after removing variables with >10% missingness. The imputation of these missing values was unsuccessful for 36 variables which were removed from the analysis. On average the 36 variables were >99·9% (s.d. ± 0·32) uniform which explains the incomplete imputation.

The average percent uniformity for the remaining 1133 variables was 89·9%. The 278 variables with >99% uniformity were removed. The final variable count in each dataset is shown in Table 2.

**Table 2.** List of asset wealth variables by variable type

Count (1–10)	Count (11–20)	Binary
Dwellings	Cooking fuel – firewood	Radio
Iron roofs	Cooking fuel – charcoal	Television
Thatch roofs	Cooking fuel – gas stove	Cupboard
Unburnt brick walls	Cooking fuel – paraffin stove	Sofa with cushions
Mud brick walls	Latrine on compound	Clock
Cement brick walls	Completely closed latrine	Wrist watch
Mud/cement walls	Partially closed latrine	Sewing machine
Earth floors	Open pit latrine	Torch (flashlight)
Cement floors	Mobile phone charger	Bicycle
Electric solar	Mobile phone	Motorbike

The total count of malaria-positive subjects was 621. The average count of malaria-positive cases per homestead was 1·50 cases and ranged from 0 to 8 with with >50% having zero positive cases. The average number of human subjects per homestead was 5·05 (s.d. ± 2·94) with a maximum of 21 people. Malaria prevalence per homestead averaged at 28·25% (s.d. ± 27·35) and the overall prevalence was 29·64% (621/2095) for the entire study.

### Creating wealth indices with PCA

One variable in the asset data, ‘number of mud walls’, was found to correlate too highly with two other asset variables, ‘number of dwellings’ and ‘number of earth floors’, and was therefore omitted from the wealth-indexing PCA. The first six PCs were used to find the subsets of variables that explained more than average amount of variance in the data. The 11 and 30 variables selected for the livestock and asset subsets, respectively, are listed in Tables 2 and 3. The first PC generated using each subset of variables was used to create the wealth indices. The TLV and the livestock wealth index were determined to be collinear and therefore provided some evidence of its validity.

**Table 3.** List of livestock wealth variables by variable type

Count	Binary
Weaned female calves	Chickens
Adult castrated male cattle	Ducks
Adult entire male cattle	
Adult female cattle	
Suckling pigs	
Weaned male pigs	
Weaned female pigs	
Sows	
Boars	
Chickens	

### Selecting predictors with elastic net-regularized penalized regression and GLM

After a total of 50 iterations of cross-validation for each *α* level, the *α* values with the lowest MAE for subsets A and B were 0·05 and 0·2, respectively. The corresponding *λ* values used in the elastic-net modelling are listed in Table 4. There were 143 variables selected out of 757 from subset A and 105 out of 626 variables from subset B. The AICs of the starting GLMs with the subset of these non-zero coefficient variables are listed in Table 4. After stepwise selection of variables the models’ AICs were reduced by 177 and 92 units for subsets A and B, respectively. Further backwards stepwise elimination based on *P* value was performed which reduced the amount of variables in the model to 22 for subset A and 25 for subset B. Five variables were found in both models. The final models’ estimates are included in Tables 5 and 6.

**Table 4.** Cross-validation, elastic net and GLM parameters

Parameter	Subset A	Subset B
Cross-validation *n*-folds	223	414
Alpha	0·05	0·2
Lambda	1·385	0·2464
Number of non-zero coefficients	143	105
Akaike's Information Criterion		
At beginning of GLM	745	1123
After step procedure	568	1031
After backwards elimination	578	1043

GLM, Generalized linear model.

**Table 5.** Subset A: Generalized linear model results*

	Estimate	s.e.	RR (95% CI)	*z* value	Pr(>|*z*|)
(Intercept)	−0·3475	0·5563	0·7065 (0·2374–2·1019)	−0·62	0·5321
Keep chickens (yes *vs*. no)	−0·6002	0·1963	0·5487 (0·3735–0·8062)	−3·06	0·0022
Travel to medical facility by *matatu*† (yes *vs*. no)	−0·7731	0·3183	0·4616 (0·2473–0·8614)	−2·43	0·0152
Last bought/acquired cattle 1–2 months age (yes *vs*. no)	−1·1209	0·4271	0·3260 (0·1411–0·7529)	−2·62	0·0087
Are cattle herded with goats or sheep? (yes *vs*. no)	−0·4025	0·1337	0·6686 (0·5145–0·8690)	−3·01	0·0026
Control worms in cattle with drench (unknown drug) (yes *vs*. no)	−0·2855	0·1313	0·7516 (0·5811–0·9722)	−2·18	0·0296
Pigs – use a worm control product when they get thin (yes *vs*. no)	−1·6077	0·7212	0·2003 (0·0487–0·8235)	−2·23	0·0258
Number of houses with brick or cement walls	−0·7013	0·3057	0·4959 (0·2724–0·9029)	−2·29	0·0218
Own a bicycle for transportation (yes *vs*. no)	0·4330	0·1858	1·5419 (1·0713–2·2192)	2·33	0·0197
Number of individuals in 5–9 years age group	1·4108	0·3342	4·0992 (2·1293–7·8918)	4·22	0·00002
Samia subgroup (yes *vs*. no)	0·5738	0·1889	1·7750 (1·2258–2·5703)	3·04	0·0024
Feeding livestock once a week (yes *vs*. no)	1·0625	0·2577	2·8936 (1·7462–4·7950)	4·12	0·00004
Used to but no longer involved with manure preparation (yes *vs*. no)	3·7715	1·5590	43·445 (2·0461–922·497)	2·42	0·0156
Human subject milks cow at least once a year (yes *vs*. no)	1·2721	0·6305	3·5683 (1·037–12·2786)	2·02	0·0436
Seek treatment for breathing problem at a hospital (yes *vs*. no)	−1·3600	0·5119	0·2567 (0·0941–0·7000)	−2·66	0·0079
Currently taking medications (yes *vs*. no)	−1·1713	0·4627	0·3100 (0·1252–0·7676)	−2·53	0·0114
Human faecal-positive for *Schistosoma mansoni* (yes *vs*. no)	−1·0352	0·4217	0·3552 (0·1554–0·8117)	−2·45	0·0141
Cattle faecal-positive *Trichuris* (whipworm) (yes *vs*. no)	0·0874	0·0361	1·0913 (1·0168–1·1713)	2·42	0·0155
High-grade cattle breed, e.g. Friesian cross (yes *vs*. no)	−1·6162	0·7112	0·1987 (0·0493–0·8007)	−2·27	0·0231
Prophylactic treatment of cattle when ticks seen (yes *vs*. no)	0·4190	0·1559	1·5204 (1·1201–2·0638)	2·69	0·0072
Average cattle skin elasticity rating (yes *vs*. no)	−0·4189	0·1809	0·6578 (0·4614–0·9377)	−2·32	0·0206
Had fever but did not seek treatment (yes *vs*. no)	0·6547	0·2636	1·9246 (1·1480–3·2263)	2·48	0·0130
Use Nambale cattle market (yes *vs*. no)	−0·6138	0·2423	0·5413 (0·3367–0·8703)	−2·53	0·0113

s.e., Standard error; RR, relative risk; CI, confidence interval.

* Number of observations = 224.

† Minibuses, station wagons, vans and pick-up trucks serve as *matatus*.

**Table 6.** Subset B: Generalized linear model results*

	Estimate	s.e.	RR (95% CI)	*z* value	Pr(>|*z*|)
(Intercept)	0·0161	0·8778	1·0162 (0·1819–5·6778)	0·02	0·9854
Number of individuals in the 15–19 years age group	0·0849	0·0405	1·0886 (1·0055–1·1785)	2·09	0·0363
Keep ducks (yes *vs*. no)	−0·2538	0·1287	0·7758 (0·6029–0·9984)	−1·97	0·0487
Experienced drought in the last 6 months (yes *vs*. no)	0·3722	0·1151	1·4509 (1·1579–1·8181)	3·23	0·0012
Keep cattle to sell as adult cattle (yes *vs*. no)	−0·2877	0·0996	0·7500 (0·6170–0·9117)	−2·89	0·0039
Use Nambale cattle market (yes *vs*. no)	−0·6991	0·2377	0·4970 (0·3119–0·7920)	−2·94	0·0033
Cattle's water collected from river – dry season (yes *vs*. no)	0·3053	0·1331	1·3570 (1·0454–1·7615)	2·29	0·0218
Pigs freely roam in the dry season (yes *vs*. no)	0·5482	0·2414	1·7301 (1·0780–2·7769)	2·27	0·0232
Waste is cooked prior to being fed to pigs (yes *vs*. no)	−0·3825	0·1595	0·6822 (0·4990–0·9325)	−2·40	0·0165
Number houses with cement floors	−0·2774	0·0777	0·7578 (0·6507–0·8824)	−3·57	0·0004
Own a bicycle for transportation (yes *vs*. no)	0·3894	0·1186	1·4761 (1·1699–1·8624)	3·28	0·0010
Altitude	−0·0015	0·0007	0·9985 (0·9971–0·9999)	−2·21	0·0273
Number of individuals in the 5–9 years age group	1·0692	0·2892	2·9130 (1·6526–5·1347)	3·70	0·0002
Number of individuals in the 10–15 years age group	1·0027	0·2760	2·7256 (1·5868–4·6816)	3·63	0·0003
Occupation – teacher (yes *vs*. no)	−4·3639	1·4921	0·0127 (0·0007–0·2371)	−2·92	0·0035
Occupation – fisherman (yes *vs*. no)	−3·7469	1·4198	0·0236 (0·0015–0·3813)	−2·64	0·0083
Occupation – none (yes *vs*. no)	1·2529	0·5319	3·5005 (1·2342–9·9285)	2·36	0·0185
Feeding livestock once a week (yes *vs*. no)	0·7506	0·2047	2·1183 (1·4182–3·1639)	3·67	0·0003
Pigs kept in buildings (yes *vs*. no)	0·8555	0·3267	2·3526 (1·2401–4·4630)	2·62	0·0088
Recent illness – abdominal pain (yes *vs*. no)	0·5050	0·2359	1·6570 (1·0436–2·6310)	2·14	0·0323
Recent illness – eye problems (yes *vs*. no)	−2·3010	0·8811	0·1002 (0·0178–0·5632)	−2·61	0·0090
Had fever and treated by chemist (yes *vs*. no)	−0·6691	0·2872	0·5122 (0·2917–0·8992)	−2·33	0·0198
Currently taking medications (yes *vs*. no)	−0·7147	0·3215	0·4893 (0·2606–0·9189)	−2·22	0·0262
Recent backache (yes *vs*. no)	−0·5276	0·2410	0·5900 (0·3679–0·9462)	−2·19	0·0286
Recent shortness of breath (yes *vs*. no)	0·8706	0·3271	2·3883 (1·2580–4·5345)	2·66	0·0078
Recent adenitis (yes *vs*. no)	−1·2650	0·6213	0·2822 (0·0835–0·9538)	−2·04	0·0418

s.e., Standard error; RR, relative risk; CI, confidence interval.

* Number of observations = 415.

## DISCUSSION

A well-defined protocol for shrinking large datasets to a manageable list of predictors has not yet been documented due to the difficultly in accommodating different needs and types of dataset. The PAZ data is a good representation of a dataset produced by many disciplines to which this methodology could be applied; it encompasses data from several different sources (biological sampling, questionnaires, direct observation), both binomial and categorical variables, many missing values, and highly correlated variables. The procedure described above successfully reduced 1376 variables to 42 predictors of malaria and produced wealth rankings for all homesteads. We believe this protocol is simple and efficient while having enough flexibility in its method to accommodate different datasets.

The steps to make a complete dataset were effective and flexible. The original dataset had an average of 8·99% missing values and after the limit of 10% missingness was applied, 89·89% of those were eliminated from the analysis. This supported the use of the 10% limit and makes the imputations process less computationally taxing. This limit could be disregarded or increased with other datasets if they can meet the requirement of missing at random. Piecewise multiple imputations by chained equations (MICE) successfully imputed the majority of variables with only five iterations. The few variables that were not completely imputed were found to be uniform in nature and would have been eliminated in the next step, i.e. the elimination of highly uniform variables, even if full imputation would have been encouraged by increasing iterations. The number of MICE iterations and the uniform limit could be adjusted according to the needs of individual dataset.

PCA successfully grouped a subset of asset and livestock variables to create wealth indices. Even though the wealth indices were not part of the final models, because of lack of statistical significance, several wealth variables were found to be significant which supports the validity of the wealth indices. The step of choosing the best *α* level for the elastic net adds to the flexibility of this protocol and will accommodate other datasets that have different numbers of correlated variables. The final GLM also has options regarding how variables are eliminated from the model, i.e. forward, backward or both directions. Finally, depending on the study's needs, one could choose an end point as the model with the lowest AIC or one only having significant variables remaining.

In future editions of this protocol, other tools could be added such as Bayesian disease mapping and network analysis. Steps to determine if missing observations are missing at random could be incorporated in addition to other model types, such as zero-inflated models, which would also add variety to its application for outcomes with low prevalence. Elastic net is a good technique for data mining of large datasets but can struggle with highly correlated variables sometimes requiring correlated variables to be removed from the model in order for other significant predictors to emerge. Exploring possible correlations >89% between variables could be performed if highly correlated variables are expected and if there was an undesirable effect on the model's output.

The proposed systematic data-mining approach resulted in the selection of 42 risk factors, a portion of which were related to exposure, wealth, or age. Increased exposure variables are those that increase time spent outside or near water (e.g. ‘own a bicycle for transportation’, ‘feeding livestock once a week’, ‘water is collected from the river for cattle in the dry season’). Homesteads that ‘keep ducks’ and/or ‘keep chickens’ were associated with lower homestead malaria prevalence, which may be a result of decreased human exposure to malaria via zooprophylaxis, in which mosquitos might feed on animals in the area, making them less likely to feed on humans [14]. Cement floors and brick or cement walls were also associated with lower homestead malaria prevalence, which may be due to a decrease in the amount of mosquitoes in the home due to physical barriers. These homestead characteristics also represent a homestead's wealth which aligns with the correlation between wealth and decreased disease incidence [2]. Other variables selected which might represent wealth include having high-grade cattle (e.g. Friesian cross) and having access to healthcare such as ‘seek treatment for breathing problem at a hospital’, ‘currently taking medications’ and ‘had fever and treated by chemist’ (in Kenya, a chemist is understood to be a healthcare professional that practises pharmacy). It has been well documented that children have the highest malaria prevalence [15]. Younger age groups (5–9, 10–14, 15–19 years) were found to be significant determinants of increased malaria diagnosis, along with variables related to being younger (e.g. ‘occupation – none’). While some of these examples are supported by previously published associations, confounders and variables not measured in this study could be factors; therefore, this approach should be viewed as more of a hypothesis-generating tool.

In conclusion, the proposed approach in which a number of statistical techniques are used including multiple imputation of missing values, wealth indexing through PCA, elastic net, and generalized linear regression models was successful in reducing a wide, sparse dataset to a more useful, simplified set of predictors for falciparum malaria infection prevalence and producing socioeconomic wealth indices. The protocol's flexibility suggests that it may be applied to other areas of epidemiology and infectious diseases and it also may serve as a hypothesis-generating tool to guide more detailed studies. In addition, we can now prioritize variables associated with malaria prevalence in the area of study and this can help the Kenyan health policy-makers prioritize their resources.
